# Assessing Metal Exposures Among Children Living in Environmental Justice Communities Near Metal Recycling Facilities in Houston, Texas

**DOI:** 10.1089/env.2022.0023

**Published:** 2024-04-19

**Authors:** Elaine Symanski, Kristina W. Whitworth, Inkyu Han, Amal Rammah, Juan Alvarez, Iman Moussa, Heyreoun An Han, Juan Flores

**Affiliations:** Center for Precision Environmental Health and Departments of Medicine and Family and Community Medicine, Baylor College of Medicine, Houston, Texas, USA.; Center for Precision Environmental Health and Department of Medicine, Baylor College of Medicine, Houston, Texas, USA.; Department of Epidemiology and Biostatistics, Temple University College of Public Health, Philadelphia, Pennsylvania, USA.; Center for Precision Environmental Health, Baylor College of Medicine, Houston, Texas, USA.; Center for Precision Environmental Health, Baylor College of Medicine, Houston, Texas, USA.; Center for Precision Environmental Health, Baylor College of Medicine, Houston, Texas, USA.; Center for Precision Environmental Health, Baylor College of Medicine, Houston, Texas, USA.; Air Alliance Houston, Houston, Texas, USA.

**Keywords:** metals, environmental exposure, children, environmental justice, urine

## Abstract

**Background:**

Children living in environmental justice (EJ) neighborhoods may be vulnerable to metal exposure from industrial facilities that are located near their homes.

**Methods:**

Working with community partners, we held 20 recruitment events and invited children aged 5–12 and their parents living in EJ communities in Houston to participate in an environmental health study. Parents completed a questionnaire about their child’s diet and behaviors and urine samples were collected from children to evaluate their metal exposure.

**Results:**

During a 4-month period, we recruited 52 out of 67 (78%) eligible parent/child dyads with 96% of children providing urine samples and 90% of questionnaires complete except for data on children’s height and weight. While urinary metal concentrations in our study population were generally similar compared with children aged 6–11 years in the 2015–2016 National Health and Nutrition Examination Survey, we observed higher levels among children who frequently ate Mexican candy, rice, or red meat, spent more time outdoors, played with cosmetics, had metal piercings, or lived in a home with smokers or where pesticides were used.

**Discussion:**

Our study was successful in recruiting children in EJ communities for the purpose of assessing urinary metal exposure and obtaining questionnaire data from parents to examine the potential sources of exposure. Except for chromium and cobalt, 14 metals were detected in more than half of children’s urine samples. We identified potential key determinants of exposure in this population that should be further examined.

**Conclusion:**

Findings point to the need for adequately powered studies among potentially vulnerable children living in EJ communities to profile metal exposures and identify important sources of these exposures.

## INTRODUCTION

Potential exposure to toxic metals is a concern in Houston, Texas, a city with the largest petrochemical complex in the world, heavily trafficked roadways, and >100 metal recycling facilities.^1,2^ These sources of metal pollution coupled to a legacy of redlining,^3^ no zoning laws,^4^ and a mixed land use policy^5^ have resulted in industrial sites being located “next door” to residential neighborhoods. Predominantly poor and largely Hispanic/Latino or African American, such neighborhoods are home to environmental justice (EJ) communities that often experience an inequitable burden of environmental exposures. In addition, Houston is prone to hurricanes and flooding,^6^ which raises the potential for redistribution of metals previously trapped in soil and sediments as was evidenced in New Orleans communities following Hurricanes Katrina and Rita.^7,8,9,10^

Heavy metals emitted from point sources may contaminate the environment and become a source of exposure for individuals living near these facilities. Children are especially at risk of exposure as they are more likely to play outdoors in bare soil areas or indoors on the floor and may ingest soil or dust through hand-to-mouth behaviors.^11^ Although the link between early life lead (Pb) and mercury (Hg) exposures and poor neurocognitive out-comes among children has been documented,^12^ the literature regarding potential neurotoxic impacts of other metals is less robust. A meta-analysis reported statistically significant associations between exposure to manganese (Mn) and decreased IQ in children^13^; another meta-analysis reported neurodevelopmental effects in children associated with exposure to arsenic (As) and Mn.^14^

Metal exposure may also result in other adverse health effects among children. For example, Mucino-Sandoval *et al.*^15^ reported associations between early life Pb exposure and markers of metabolic syndrome, including increased waist circumference and high-density lipoprotein cholesterol, among children aged 6 to 12. In addition, in a study using the National Health and Nutritional Examination Survey (NHANES) data, adverse associations were reported between exposures to Mn and Pb and pulmonary function among children aged 6–17 years^16^ and another cross-sectional study of impaired pulmonary function detected inverse associations with copper (Cu), vanadium (V), and Mn exposure among asthmatic children ages 7 to 15 in Chicago, Illinois.^17^

While prior studies have reported elevated urinary levels of metals among children living close to petrochemical and gas refineries,^18^ waste incinerators,^19,20^ waste sites,^21^ or areas where copper smelting,^22,23^ metal mining,^24,25^ or metallurgical industries^26^ were located, we are not aware of studies that focused on metal exposure among children living near metal recycling plants. Furthermore, there are barriers in recruiting members of disadvantaged groups in epidemiologic studies^27,28^ and in collecting biospecimens^29^ that result from mistrust, a perceived lack of benefit,^30^ and lack of interest.^31^

We leveraged an ongoing community-engaged project called Metal Air Pollution Partnership Solutions (MAPPS)^32,33^ to launch the Children’s Health and Research on Metals (CHaRM) study with a primary objective to characterize metal exposure via biomonitoring among children living in MAPPS neighborhoods. Given the potential concern about the redistribution of metals in soil following intense flooding events, our study was additionally focused on children who lived in these neighborhoods during Hurricane Harvey (2017) and remained in the same house since that time.

## MATERIALS AND METHODS

The MAPPS and CHaRM studies were approved by the Institutional Review Boards at Baylor College of Medicine and The University of Texas Health Science Center at Houston. MAPPS was designed to address resident concerns about metal aerosol emissions from metal recycling facilities in four low-income, predominantly Hispanic and African American, communities in Houston (i.e., Magnolia Park [two locations], South Park, and Fifth Ward/Northside).^34,35^

Key elements of the MAPPS project included the following: (1) promoting community engagement through a Community Advisory Board (CAB) that comprised residents, metal recycling representatives, and the research team (academics, Houston Health Department officials, and members of an EJ advocacy group); (2) outdoor air sampling for metals in the four neighborhoods; (3) health risk assessments based on the air monitoring results; (4) assessment of environmental health perceptions and needs; (5) translating and disseminating study findings; and (6) developing and implementing a public health action plan based on the results.

Following Hurricane Harvey, we designed the CHaRM study to (1) collect soil samples for measuring and comparing metal concentrations between neighborhoods and (2) recruit parents and children in MAPPS neighborhoods for a biomonitoring study of children’s metal exposures and identify potential sources of children’s metal exposure using questionnaire data. Results from the assessment of the soil samples have been published previously;^36^ herein we report on the biomonitoring component of the CHaRM study.

Eligibility was based on the following criteria: (1) the parent was 18 years or older; (2) the child was aged 5–12 years and living with the parent (the lower age limit was imposed due to logistical considerations in collecting urine samples, and the upper age limit to exclude adolescents whose behaviors vary from those of younger children); (3) self-reported residence within ~ 1 mile (1.6 km) of one of the four metal recycling facilities in MAPPS neighborhoods, based on visual inspection of a map; and (4) residence in the same household since Hurricane Harvey. Parents were provided a $20 gift card and school supplies as a measure of appreciation for participation.

The study team consulted with the CAB, along with residents who completed an Environmental Health Leadership training workshop, a component of the public health action plan developed in the parent study. We held individual and group meetings to seek input on biospecimen collection, promotional materials, and recruitment strategies. Relying on urine sampling was recommended considering acceptability, convenience, and cost. Suggestions were provided on partners, messaging, venues, timing of promotion and recruitment events. Bilingual CAB members provided feedback on the Spanish versions of the questionnaire and recruitment flyers.

Upon enrollment, a staff member obtained informed consent from the parent and assent from the child. Questionnaires were administered in English or Spanish and asked about sociodemographic characteristics; height and weight of the child; frequency of consumption of selected food items in the past 3 months (never or <1 time per month; 1–3 times per month; 1 time per week; >1 time per week but not every day; 1 time per day; >1 time per day); average time spent playing outdoors per week (never; <2.5 hours; 2.5 to <5 hours; 7 or more hours); whether the child played with or used crayons, pottery, metal jewelry, cosmetics, artist’s paint, painted toys, sunscreen; usual behaviors of the child (e.g., handwashing, removing shoes while inside the house); characteristics of the home environment, including any smoking inside the home, the presence of pets, and use of pesticides in or around the home; and flooding and damage to the home during or after Hurricane Harvey.

Questions related to diet were informed by the NHANES diet behavior and nutrition questionnaire^37^ and the Harvey-related questions were modified versions of questions from the WTC-Sandy questionnaire.^38^

We provided instructions to each child (or to a parent if they were assisting) on how to provide the urine sample in a sterile orange-top polypropylene 4.5-ounce cup labeled with a study ID. After collection, the sample was stored in a cooler on ice. At the end of each recruitment event, samples were transported to the laboratory, where they were stored at −20°C. All urine samples were analyzed for metals within 3 months of collection. An inductively coupled plasma mass spectrometer (ICP/MS 7500; Agilent Technologies, Palo Alto, CA) was used for quantification of 16 metals: antimony (Sb), total As, barium (Ba), cadmium (Cd), cobalt (Co), Cu, chromium (Cr), iron (Fe), Pb, Mn, nickel (Ni), selenium (Se), strontium (Sr), thallium (Tl), V, and zinc (Zn). Standard calibration curves ranging from 0.1 to 1000 ng/mL were constructed.

For each metal, we determined the limit of detection (LOD) as three times the standard deviation of seven replicate analyses of the lowest standard solution (1 ng/mL) for all metals except Fe (10 ng/mL). The LODs (ng/mL) were as follows: As (0.181); Ba (0.111); Cd (0.100); Co (0.178); Cr (0.195); Cu (0.253); Fe (18.079); Mn (0.188); Ni: (0.225); Pb (0.109); Sb (0.098); Se (0.802); Sr (1.039); Tl (0.090); V (0.090); and Zn (0.266). Measured metal concentrations in empty urine containers were subtracted from the measured metal concentrations in urine samples. Urinary metal measurements below the LOD were replaced by $LOD∕\sqrt{2}$.^39^ Urinary creatinine was measured to report urinary metal concentrations with creatinine adjustment (μg/g creatinine).

We computed participation rates by neighborhood, along with metrics regarding adherence to the protocol, including completeness of questionnaires and provision of urine samples. We computed body mass index (BMI) (kg/m^2^) from reported height (inches) and weight (pounds) and categorized it into age- and sex-specific percentiles as per CDC guidelines.^40^ We geocoded the child’s address at enrollment using ArcGIS Pro (Version 2.7.3; ESRI, Red-lands, CA) and evaluated whether households fell within the 1-mile buffers surrounding each metal recycling facility. We computed geometric means (GM) and confidence intervals (95% CIs), along with selected percentiles of the distributions, of the urinary concentrations of metals, and conducted Spearman correlation analyses between individual metal compounds. Finally, before computing GM metal concentrations stratified by sociodemographic and other characteristics, we collapsed levels of some of these variables because of sparse data. All statistical analyses were performed using SAS version 9.4 (SAS Institute, Cary, NC).

## RESULTS

From April to November 2019, and with assistance from community partners, we promoted the study by distributing bilingual flyers in-person and through e-mail at community centers, schools, churches, libraries, clinics, apartment complexes, and local businesses throughout the four neighborhoods. We also participated in community events where bilingual staff conducted preliminary screening of 389 families (152 in Northside/Fifth Ward; 120 in Magnolia Park, and 117 in South Park) to determine eligibility and collected contact information from those interested to send reminders about recruitment events. We then invited parents and their children to participate in our study from August to November 2019 at stand-alone recruitment events, back-to-school events, health fairs and during pickup of children who attended after-school programs (*n* = 20 events).

As shown in Table 1, we screened 91 parent/child pairs, of which 67 (74%) met the eligibility criteria and 52 (78%) enrolled. Two children from the same family were enrolled at separate enrollment events, and hence, the total number of parents is one less than the number of enrolled children. Reasons for not participating included lack of interest in research, distrust of the use of study results, a lack of awareness of the impact of metal exposures on children’s health, or greater interest in participating in other activities where recruitment was taking place. All but two children provided urine samples and the questionnaires were largely completed except for questions on the height and weight of children. After geocoding residential addresses, we determined that 46% of participants’ homes fell within the 1-mile (1.6 km) buffers of metal recycling facilities.

The mean (SD) age of children in this study was 9 (1.9) years (Table 2). Just over half of the children were female (52%), with 85% identified by their parents as Hispanic. We could not compute BMI for nearly half (46%) of the children because of missing data on height and/or weight. Among the 28 children for whom we had height and weight data, 19 (37%) were categorized as overweight/obese. Most parents had an annual household income below $25,000 (57%) and reported high school as their highest level of education (55%). Sixteen (31%) families experienced flooding during Hurricane Harvey.

Table 3 depicts the distributions of children’s urinary metal concentrations. Six metals (As, Ba, Cu, Se, Sr, and Zn) were detected in 100% of the urine samples. In contrast, 31 (62%) and 37 (74%) children had non-detectable urinary concentrations of Co and Cr, respectively, and these metals were excluded from further analyses. Strong Spearman correlations (>0.7) were detected for the following metal pairs: Fe with Cu; Pb with Sb; Mn with Ba and Fe; Se with Cu; Sr with Ba, Fe, and Mn; Tl with Cu and Se; V with Ba, Cu, Fe, Mn, and Sr; and Zn with Cd (Supplementary Table S1).

GM urinary metal concentrations were higher for almost all metals except Cd and Ni (although with overlapping CIs) among the relatively few participants who lived in homes where smoking was reported (*n* = 5) compared with children who lived in nonsmoking households (Table 4). There was no evidence of differences in metal exposures based on whether children’s homes flooded during Hurricane Harvey. Similarly, there were no discernible patterns in exposure for most behavioral and household characteristics (Table 5). However, for most metals, we observed higher urinary metal concentrations among children who played with cosmetics or who lived in homes where pesticides were used. Except for Zn and Cd, we also observed higher levels of urinary metal concentrations among children who, on average, played outdoors at least 5 hours per week compared with those who spent less time in outdoor play.

Finally, we observed a 1.5-fold difference in the urinary concentration levels of Sr in children with piercings [GM = 41.84 (26.74–65.45) μg/g creatinine] compared with those without piercings [GM = 28.94 (21.25–39.41) μg/g creatinine].

Table 6 shows potential dietary exposure to metals among study participants and a few patterns emerged. For example, compared with children who ate rice less than daily, children who ate rice at least once per day had higher levels of multiple metals with the most notable difference for As; GM = 6.59 (4.05–10.72) μg/g creatinine compared with GM = 4.57 (3.47–6.01) μg/g creatinine. Children who consumed candy from Mexico at least weekly had higher urinary levels of all metals than those who consumed Mexican candy less frequently. Finally, we observed higher urinary concentrations of most metals among children with frequent consumption of red meat, whereas the opposite pattern was observed for fish.

## DISCUSSION

This is the first study, to our knowledge, to examine metal exposure using biomonitoring among children living in EJ neighborhoods near metal recycling facilities. Where comparisons could be made, GM levels for As, Ba, Co, Pb, Sr, and Tl were similar to or lower in our study population than the 2015–2016 NHANES for children ages 6 to 11 years.^41^ However, GM (95% CI) levels of Sb were higher in our study [0.13 (0.10–0.17) μg/g creatinine] than reported in NHANES [0.08 (0.08–0.10) μg/g creatinine]. Furthermore, GM concentrations of Sb, Ba, Cd, Cr, Co, Cu, Pb, Mn, Ni, and V were similar to or lower than those reported in previous biomonitoring studies among children.^42,43,44,45,46,47^

However, urinary As levels [GM = 4.99 (3.94–6.31) μg/g creatinine] were higher in our study than previously reported among children aged 6 to 12 years living near gas and petrochemical industries in Asaluyeh, Iran [GM = 2.63 (1.96–3.69) μg/g creatinine]^48^ or children aged 5 to 17 (GM = 1.60 μg/g creatinine; 95% CI not reported)^49^ or 6 to 9 years [GM = 2.44 (1.79–3.53) μg/g creatinine]^50^ living in the Ria de Huelva in a highly industrialized area in southwest Spain. In contrast, urinary As levels in our study were considerably lower than among children living near a metallurgical industry in San Luis Potosí, Mexico [GM = 47.5 (25.0–50.0) μg/g creatinine],^51^ or near major roadways in Lahore, Pakistan [GM = 50.9 (35.5–76.5) μg/g creatinine].^52^ In this latter study in Pakistan, urinary Se levels [GM = 25.4 (16.8–40.6) μg/g creatinine] were lower than in our study [GM = 53.65 (45.41–63.40) μg/g creatinine].

Our exploration of determinants of children’s metal exposures was limited by sample size, and thus, we did not perform statistical comparisons. However, a few differences emerged in comparing urinary metal concentrations for a few of the questionnaire variables. Consistent with studies that have detected metals in tobacco, cigarette paper, filters and cigarette smoke,^53^ Mexican candy,^54^ and rice,^55^ we found that urinary levels of most metals were higher among children living in households where someone smoked or those who frequently consumed Mexican candy and rice. We also observed higher levels for some metals among children who frequently played outdoors or with cosmetics. While the former is consistent with the long-recognized impact of children’s activity patterns on environmental exposures,^56^ the use of cosmetics (or other personal care products) as a potential source of metal exposure among children warrants future investigation.

The ideal biomarker should be sensitive, specific, biologically relevant, and practical.^57^ In addition, when dealing with children, biomarkers should be noninvasive and have values available for comparison with the general population.^58^ In our study, we chose to collect urine specimens given the relative ease and low cost of collecting these samples, and we were successful in obtaining samples from all but two children. While our results suggest that urine sampling could be easily adapted in future epidemiological studies, we recognize that for some metals (e.g., Pb), blood is the preferred biological matrix.^59^ Second, metals in urine may reflect either recent (e.g., As)^60^ or long-term (e.g., Cd)^61^ exposure. Hence, selecting an optimal biomarker depends heavily on the research question being asked.

Our participation rates (ranging from 50% to 85% by neighborhood) were likely buoyed by our partners who helped develop promotion and recruitment strategies, highlighting the importance of engaging community members in an early stage of research activities.^62,63^ Notwithstanding limitations with snowball sampling,^64^ the most fruitful collaborations were with staff at community centers and schools who became visible promoters by personally inviting center members to recruitment events. The most successful events were those integrated with health fairs or back-to-school drives as these were familiar to and conveniently scheduled for residents, or where there were no competing demands (such as childcare) or limited time, both potential barriers to participation.^65^

Barriers to recruitment that may have impacted our study include participants’ lack of experience with research studies^66^ and lack of knowledge about, and therefore interest in, metal exposures. Hence, an environmental health literacy campaign focusing on children’s metal exposure held before and during field campaigns may have increased participation. With only three bilingual (Spanish and English) staff members, we were restricted in reaching the Spanish-speaking population. We noted positive effects of having one of our MAPPS CAB members, an African American civic leader, present at enrollment events and we recognize that having African American field staff would likely have increased participation among the African American residents.^67,68^ Notwithstanding these challenges, recruitment of hard-to-reach populations is labor- and time-intensive, as evidenced from a recent report of nearly 100 predominantly Hispanic children living in a disadvantaged neighborhood close to an industrial facility who were recruited over a 12-month period.^69^

## CONCLUSION

While urinary concentrations of most metals in our study were similar to or lower than levels reported in previous studies, our findings point to potentially important sources of exposure that warrant further investigation. With critical community engagement underpinning our successes, our study also demonstrated feasibility in administering questionnaires to ascertain information about sociodemographic factors and potential exposure sources and in collecting urine samples from children living in EJ neighborhoods. Future adequately powered studies are needed to enhance our understanding of the magnitude and extent of variability, as well as identify subgroups who may face elevated risks of exposure because of what they eat, how they play, and where they live or go to school.

## Supplementary Material

Env Justice 2014 17:2, 128-142 (supplementary material)

## Figures and Tables

**Table 1.** Feasibility Metrics, Children’s Health and Research on Metals Study, Houston, Texas, August–November 2019

	Northside/ Fifth Ward	Magnolia Park (East and West)	South Park	Total, N(%)
Parent/child pairs screened,^a^ *n*	10	58	23	91
Parent/child pairs eligible, *n*	10	40	17	67
Parent/child pairs enrolled, *n* (%)	5 (50)	34 (85)	13 (77)	52^b^ (78)
Questionnaire data, *n* (%)				
Complete questionnaire	4 (80)	15 (44)	5 (38)	24 (46)
Complete questionnaire except height and weight	5 (100)	32 (94)	10 (77)	47 (90)
Missing questionnaire data other than height and weight^c^	0 (0.0)	2 (6)	3 (23)	5 (10)
Accurate reporting of residence within a 1-mile buffer, *n* (%)	2 (40.0)	16 (47.1)	6 (46.2)	24 (46)
Urine samples, *n* (%)	5 (100)	33 (97)	12 (92)	50 (96)

a Families or individuals that were screened during recruitment events.

b Fifty-one parents/guardians as two children were enrolled from the same family.

c Missing data included responses to “other vitamin intake” and “type of flooding found in home (carpet or other).”

**Table 2.** Sociodemographic Profile of Parent/Child Dyads (*n* = 52)^a^ in the Children’s Health and Research on Metals Study, Houston, Texas, August–November 2019

	N (%)
Child’s age (years)	
5–9	26 (50)
10–12	26 (50)
Child’s sex	
Male	25 (48)
Female	27 (52)
Child’s race and ethnicity	
Non-Hispanic white	1 (2)
Non-Hispanic black	6 (12)
Hispanic	44 (85)
Does not know	1 (2)
Child’s BMI^b^	
Underweight/normal	9 (17)
Overweight/obese	19 (37)
Missing	24 (46)
Smoking in the home	
Yes	5 (10)
No	46 (90)
Parent’s sex	
Male	1 (2)
Female	50 (98)
Parent’s educational attainment	
Elementary/middle school (K-8)	13 (25)
High school (grades 9–12)	28 (55)
At least some college	10 (20)
Parent’s civil/marital status	
Married/cohabiting	30 (59)
Single/widowed/divorced	21 (41)
Parent’s annual household income	
<$25,000	29 (57)
≥$25,000 to <$50,000	17 (33)
≥$50,000 to <$100,000	3 (6)
Does not know	2 (4)
Home flooded during Harvey	
Yes	16 (31)
No	35 (69)

a 51 parents/guardians as two children were enrolled from the same family.

b BMI was categorized based on age- and sex-specific percentiles. Underweight/normal: BMI <85th percentile; overweight/obese: BMI ≥85th percentile.

BMI, body mass index.

**Table 3.** Distribution of Creatinine-Adjusted Urinary Metal Concentrations (μg/g Creatinine) Among 50 Children Ages 5–12, Children’s Health and Research on Metals Study, Houston, Texas, August–November 2019

	N < *LOD (%)*	*Mean (95% CI)*	*GM (95% CI)*	*Percentiles*						
				*5%*	*10%*	*25%*	*50%*	*75%*	*90%*	*95%*
Antimony (Sb)	1 (2)	0.19 (0.14–0.25)	0.13 (0.10–0.17)	0.04	0.05	0.07	0.10	0.23	0.46	0.70
Arsenic (As)	0 (0)	7.05 (5.18–8.92)	4.99 (3.94–6.31)	1.44	1.80	2.62	4.53	8.62	15.41	21.02
Barium (Ba)	0 (0)	0.80 (0.59–1.01)	0.55 (0.43–0.71)	0.12	0.15	0.31	0.55	0.92	1.78	2.59
Cadmium (Cd)	9 (18)	0.07 (0.06–0.08)	0.06 (0.05–0.07)	0.02	0.03	0.03	0.05	0.11	0.14	0.17
Chromium (Cr)	37 (74)	0.16 (0.04–0.28)	0.09 (0.07–0.11)	0.03	0.04	0.05	0.08	0.13	0.18	0.24
Cobalt (Co)	31 (62)	0.16 (0.06–0.27)	0.09 (0.07–0.12)	0.03	0.04	0.05	0.09	0.14	0.21	0.48
Copper (Cu)	0 (0)	1.98 (1.55–2.40)	1.51 (1.22–1.87)	0.49	0.56	0.90	1.35	2.65	4.28	4.61
Iron (Fe)	4 (8)	13.49 (10.68–16.29)	10.19 (8.16–12.73)	2.52	3.68	5.23	9.75	20.66	28.46	31.08
Lead (Pb)	4 (8)	0.33 (0.18–0.48)	0.15 (0.10–0.20)	0.03	0.04	0.07	0.11	0.27	0.97	1.71
Manganese (Mn)	11 (22)	0.13 (0.10–0.16)	0.10 (0.08–0.12)	0.03	0.04	0.05	0.10	0.18	0.25	0.30
Nickel (Ni)	15 (30)	0.54 (0.31–0.77)	0.27 (0.19–0.37)	0.07	0.07	0.11	0.21	0.58	1.28	2.44
Selenium (Se)	0 (0)	63.05 (52.89–73.20)	53.65 (45.41–63.40)	21.14	25.11	36.30	51.32	85.13	110.36	140.92
Strontium (Sr)	0 (0)	47.83 (37.40–58.27)	34.03 (26.31–44.03)	5.42	10.74	19.45	34.68	76.01	100.47	126.76
Thallium (Tl)	1 (2)	0.13 (0.10–0.17)	0.10 (0.08–0.12)	0.03	0.05	0.07	0.09	0.15	0.28	0.32
Vanadium (V)	1 (2)	0.11 (0.09–0.13)	0.09 (0.07–0.11)	0.03	0.04	0.05	0.07	0.16	0.21	0.27
Zinc (Zn)	0 (0)	206.13 (160.38–251.88)	157.68 (128.15–194.02)	53.03	70.99	90.98	119.70	302.68	400.69	582.10

CI, confidence interval; GM, geometric mean; LOD, limit of detection.

**Table 4.** Sociodemographic Breakdown of Geometric Mean (95% Confidence Interval) Urinary Metal Concentrations (μg/g Creatinine) Among 50 Children, Ages 5–12, Children’s Health and Research on Metals Study, Houston, TX, August–November 2019

	n	*Antimony*	*Arsenic*	*Barium*	*Cadmium*	*Copper*	*Iron*	*Lead*
Age (years)								
5–9	24	0.14 (0.10–0.20)	5.76 (4.23–7.85)	0.42 (0.29–0.61)	0.05 (0.04–0.07)	1.15 (0.85–1.54)	8.44 (5.84–12.19)	0.17 (0.11–0.28)
10–12	26	0.12 (0.08–0.18)	4.36 (3.03–6.28)	0.71 (0.51–1.00)	0.06 (0.04–0.08)	1.96 (1.47–2.61)	12.12 (9.27–15.84)	0.12 (0.07–0.20)
BMI								
Underweight/normal	9	0.14 (0.06–0.31)	3.42 (1.88–6.22)	0.56 (0.32–0.98)	0.06 (0.03–0.10)	1.37 (0.76–2.49)	10.05 (5.81–17.39)	0.12 (0.06–0.26)
Overweight/obese	19	0.14 (0.09–0.21)	4.58 (3.30–6.35)	0.60 (0.43–0.82)	0.06 (0.05–0.08)	1.60 (1.12–2.27)	11.85 (7.80–17.99)	0.16 (0.09–0.29)
Missing	22	0.12 (0.08–0.18)	6.26 (4.16–9.43)	0.51 (0.31–0.85)	0.05 (0.04–0.07)	1.50 (1.06–2.14)	8.99 (6.46–12.52)	0.14 (0.08–0.25)
Sex								
Male	24	0.14 (0.10–0.21)	4.71 (3.31–6.71)	0.50 (0.36–0.70)	0.06 (0.04–0.07)	1.47 (1.15–1.87)	10.03 (7.19–13.99)	0.19 (0.10–0.34)
Female	26	0.12 (0.09–0.17)	5.25 (3.75–7.36)	0.61 (0.41–0.90)	0.06 (0.04–0.08)	1.56 (1.08–2.24)	10.34 (7.50–14.25)	0.12 (0.08–0.16)
Race/ethnicity								
Black	5	0.28 (0.12–0.68)	4.04 (1.22–13.37)	0.33 (0.14–0.79)	0.06 (0.02–0.19)	1.26 (0.62–2.56)	9.08 (2.94–28.07)	0.18 (0.04–0.73)
Hispanic	43	0.11 (0.09–0.15)	5.05 (3.90–6.54)	0.58 (0.43–0.76)	0.05 (0.04–0.07)	1.50 (1.18–1.90)	9.83 (7.78–12.42)	0.13 (0.09–0.18)
Parent’s annual household income								
<25,000	29	0.12 (0.08–0.16)	5.74 (4.08–8.06)	0.51 (0.36–0.73)	0.05 (0.04–0.07)	1.30 (0.98–1.73)	8.43 (6.33–11.23)	0.15 (0.09–0.23)
≥25,000	19	0.14 (0.10–0.21)	4.03 (2.83–5.74)	0.58 (0.38–0.89)	0.06 (0.04–0.08)	1.75 (1.22–2.51)	12.88 (8.76–18.94)	0.14 (0.08–0.24)
Smoking in the home								
Yes	5	0.16 (0.05–0.53)	5.56 (3.90–7.93)	0.61 (0.14–2.67)	0.05 (0.02–0.12)	1.55 (0.35–6.78)	10.74 (3.07–37.62)	0.17 (0.05–0.50)
No	45	0.13 (0.10–0.17)	4.93 (3.80–6.40)	0.55 (0.42–0.71)	0.06 (0.05–0.07)	1.51 (1.22–1.87)	10.13 (8.04–12.76)	0.14 (0.10–0.21)
Home flooded during Harvey								
No	33	0.14 (0.10–0.18)	4.84 (3.65–6.43)	0.61 (0.45–0.83)	0.06 (0.05–0.07)	1.53 (1.15–2.04)	10.81 (8.21–14.24)	0.15 (0.10–0.23)
Yes	17	0.12 (0.07–0.21)	5.28 (3.32–8.39)	0.45 (0.28–0.73)	0.05 (0.03–0.07)	1.47 (1.05–2.07)	9.08 (6.00–13.74)	0.13 (0.07–0.25)
Neighborhood								
Northside/Fifth Ward	5	0.29 (0.07–1.16)	4.30 (2.22–8.31)	1.08 (0.33–3.54)	0.06 (0.03–0.14)	2.60 (1.23–5.51)	13.55 (5.15–35.68)	0.23 (0.07–0.77)
South Park	12	0.17 (0.10–0.28)	4.39 (2.68–7.18)	0.48 (0.31–0.75)	0.06 (0.04–0.10)	1.49 (0.95–2.33)	10.48 (6.12–17.94)	0.16 (0.08–0.32)
Magnolia Park	33	0.11 (0.08–0.14)	5.34 (3.90–7.33)	0.53 (0.38–0.73)	0.05 (0.04–0.07)	1.40 (1.07–1.85)	9.66 (7.34–12.72)	0.13 (0.08–0.21)
	n	*Manganese*	*Nickel*	*Selenium*	*Strontium*	*Thallium*	*Vanadium*	*Zinc*
Age (years)								
5–9	24	0.08 (0.06–0.12)	0.24 (0.16–0.36)	52.75 (43.56–63.87)	29.14 (19.16–44.33)	0.09 (0.07–0.11)	0.07 (0.05–0.09)	136.3 (104.4–177.9)
10–12	26	0.11 (0.09–0.15)	0.29 (0.17–0.48)	54.51 (41.12–72.24)	39.28 (28.32–54.47)	0.12 (0.08–0.16)	0.11 (0.08–0.14)	180.4 (130.6–249.1)
BMI								
Underweight/normal	9	0.09 (0.06–0.15)	0.24 (0.09–0.560)	41.51 (23.54–73.19)	34.74 (14.75–81.80)	0.08 (0.04–0.14)	0.08 (0.05–0.14)	142.1 (82.0–246.2)
Overweight/obese	19	0.10 (0.07–0.15)	0.28 (0.18–0.45)	59.73 (46.88–76.11)	38.94 (26.76–56.67)	0.10 (0.07–0.14)	0.09 (0.06–0.12)	173.2 (124.3–241.3)
Missing	22	0.10 (0.07–0.14)	0.27 (0.15–0.46)	54.32 (41.92–70.39)	30.04 (19.78–45.64)	0.12 (0.08–0.16)	0.09 (0.06–0.12)	151.8 (106.8–215.7)
Sex								
Male	24	0.09 (0.07–0.13)	0.28 (0.16–0.47)	53.83 (42.45–68.26)	31.07 (22.08–43.72)	0.11 (0.08–0.15)	0.08 (0.06–0.11)	143.7 (107.4–192.3)
Female	26	0.10 (0.08–0.13)	0.25 (0.17–0.38)	53.49 (41.63–68.73)	37.02 (24.77–55.34)	0.09 (0.07–0.12)	0.09 (0.07–0.12)	171.8 (126.0–234.3)
Race/ethnicity								
Black	5	0.10 (0.04–0.25)	0.40 (0.07–2.34)	57.23 (28.33–115.62)	13.01 (2.83–59.88)	0.10 (0.03–0.36)	0.09 (0.03–0.28)	235.4 (71.3–777.5)
Hispanic	43	0.09 (0.08–0.12)	0.24 (0.17–0.33)	52.24 (43.40–62.87)	36.78 (28.59–47.33)	0.10 (0.08–0.12)	0.08 (0.07–0.10)	148.9 (120.1–184.5)
Parent’s annual household income								
<25,000	29	0.09 (0.06–0.12)	0.28 (0.18–0.42)	48.89 (38.74–61.71)	28.94 (20.92–40.03)	0.10 (0.07–0.13)	0.08 (0.06–0.11)	153.6 (117.7–200.5)
≥25,000	19	0.11 (0.08–0.16)	0.24 (0.14–0.43)	59.16 (45.19–77.44)	42.34 (26.25–68.28)	0.10 (0.07–0.14)	0.09 (0.07–0.13)	161.1 (109.6–236.8)
Smoking in the home								
Yes	5	0.11 (0.03–0.33)	0.20 (0.08–0.49)	59.61 (22.78–156.00)	39.15 (7.79–196.84)	0.11 (0.05–0.21)	0.10 (0.03–0.29)	149.7 (78.4–285.5)
No	45	0.10 (0.08–0.12)	0.27 (0.19–0.39)	53.03 (44.65–62.99)	33.51 (25.80–43.52)	0.10 (0.08–0.13)	0.09 (0.07–0.11)	158.6 (126.5–198.9)
Home flooded during Harvey								
No	33	0.10 (0.07–0.12)	0.26 (0.18–0.39)	54.66 (43.81–68.19)	39.19 (28.53–53.83)	0.10 (0.08–0.13)	0.09 (0.07–0.11)	154.1 (118.4–200.6)
Yes	17	0.10 (0.06–0.16)	0.27 (0.14–0.51)	51.76 (39.52–67.79)	25.88 (16.40–40.86)	0.10 (0.07–0.14)	0.08 (0.05–0.12)	164.9 (113.8–238.9)
Neighborhood								
Northside/Fifth Ward	5	0.17 (0.09–0.33)	0.31 (0.12–0.83)	58.66 (32.77–104.99)	49.66 (14.71–167.65)	0.12 (0.06–0.25)	0.16 (0.08–0.28)	136.5 (80.1–232.6)
South Park	12	0.10 (0.07–0.17)	0.25 (0.12–0.52)	55.07 (39.66–76.46)	26.21 (13.59–50.56)	0.10 (0.05–0.18)	0.09 (0.05–0.15)	200.1 (120.9–331.0)
Magnolia Park	33	0.09 (0.07–0.11)	0.26 (0.17–0.40)	52.44 (41.82–65.74)	35.34 (26.16–47.74)	0.10 (0.08–0.13)	0.08 (0.06–0.10)	147.8 (113.7–192.2)

**Table 5.** Geometric Mean (95% Confidence Interval) Urinary Metal Concentrations (μg/g Creatinine) by Behavioral Characteristics^a^ Among 50 Children, Ages 5–12, Children’s Health and Research on Metals Study, Houston, TX, August–November 2019

		Antimony	Arsenic	Barium	Cadmium	Copper	Iron	Lead
Played with metal jewelry								
No	28	0.13 (0.09–0.18)	5.35 (3.72–7.71)	0.53 (0.37–0.77)	0.05 (0.04–0.07)	1.43 (1.05–1.93)	10.12 (7.54–13.59)	0.14 (0.09–0.23)
Yes	22	0.14 (0.09–0.20)	4.56 (3.39–6.13)	0.59 (0.41–0.84)	0.06 (0.04–0.08)	1.63 (1.18–2.25)	10.28 (7.11–14.85)	0.15 (0.09–0.25)
Played with painted toys								
No	31	0.13 (0.09–0.18)	5.04 (3.67–6.93)	0.58 (0.43–0.79)	0.06 (0.05–0.07)	1.56 (1.16–2.11)	10.65 (8.00–14.19)	0.15 (0.10–0.24
Yes	19	0.13 (0.09–0.20)	4.90 (3.37–7.14)	0.51 (0.32–0.81)	0.05 (0.04–0.08)	1.44 (1.06–1.95)	9.47 (6.43–13.95)	
Played with cosmetics								0.13 (0.07–0.23)
No	29	0.13 (0.09–0.18)	4.68 (3.41–6.43)	0.51 (0.36–0.72)	0.05 (0.04–0.07)	1.34 (1.04–1.72)	9.10 (6.67–12.40)	0.15 (0.09–0.25)
Yes	21	0.14 (0.09–0.20)	5.44 (3.73–7.94)	0.63 (0.43–0.92)	0.06 (0.05–0.09)	1.79 (1.21–2.64)	11.92 (8.55–16.61)	0.14 (0.10–0.21)
Played with/wore sunscreen								
No	19	0.15 (0.10–0.24)	5.76 (3.71–8.94)	0.75 (0.47–1.18)	0.07 (0.05–0.10)	2.12 (1.53–2.94)	13.15 (9.78–17.68)	0.15 (0.09–0.26)
Yes	31	0.12 (0.09–0.16)	4.57 (3.44–6.06)	0.46 (0.34–0.62)	0.05 (0.04–0.06)	1.23 (0.94–1.61)	8.71 (6.40–11.87)	0.14 (0.09–0.22)
Have piercings								
No	28	0.13 (0.09–0.19)	5.43 (3.85–7.65)	0.48 (0.34–0.67)	0.05 (0.04–0.07)	1.41 (1.08–1.85)	9.15 (6.75–12.40)	0.17 (0.10–0.29)
Yes	22	0.13 (0.09–0.19)	4.48 (3.21–6.25)	0.66 (0.44–0.99)	0.06 (0.04–0.08)	1.65 (1.15–2.39)	11.68 (8.27–16.50)	0.12 (0.08–0.17)
Played with crayons								
No	7	0.09 (0.03–0.25)	3.14 (1.31–7.53)	0.75 (0.38–1.51)	0.04 (0.03–0.08)	1.59 (0.94–2.69)	10.02 (5.04–19.93)	0.09 (0.03–0.29)
Yes	43	0.14 (0.11–0.18)	5.38 (4.21–6.86)	0.53 (0.40–0.70)	0.06 (0.05–0.07)	1.50 (1.18–1.91)	10.22 (7.99–13.06)	0.16 (0.11–0.22)
Played with artist paint								
No	23	0.12 (0.08–0.17)	5.44 (3.72–7.97)	0.49 (0.31–0.77)	0.06 (0.04–0.08)	1.42 (1.01–2.00)	10.39 (7.38–14.62)	0.13 (0.08–0.21)
Yes	27	0.14 (0.10–0.21)	4.63 (3.39–6.32)	0.61 (0.46–0.82)	0.05 (0.04–0.07)	1.59 (1.19–2.13)	10.02 (7.33–13.71)	0.16 (0.10–0.26)
Played with ceramics								
No	45	0.14 (0.10–0.18)	5.07 (3.98–6.47)	0.52 (0.40–0.68)	0.06 (0.05–0.07)	1.52 (1.20–1.93)	10.37 (8.19–13.14)	0.16 (0.11–0.22)
Yes	5	0.08 (0.03–0.24)	4.29 (1.12–16.37)	0.92 (0.37–2.30)	0.05 (0.02–0.11)	1.41 (0.88–2.27)	8.68 (3.15–23.91)	0.07 (0.02–0.22)
Frequency playing outside (hrs/wk)								
Never to <2.5	15	0.13 (0.09–0.18)	3.97 (2.58–6.11)	0.47 (0.31–0.72)	0.06 (0.04–0.08)	1.47 (1.05–2.05)	10.69 (7.28–15.71)	0.10 (0.06–0.18)
2.5 to <5	16	0.10 (0.06–0.15)	4.63 (3.16–6.79)	0.46 (0.28–0.75)	0.05 (0.03–0.07)	1.39 (0.95–2.03)	7.38 (4.62–11.80)	0.10 (0.06–0.16)
≥5	19	0.17 (0.11–0.28)	6.35 (4.06–9.92)	0.73 (0.46–1.14)	0.06 (0.05–0.09)	1.67 (1.08–2.58)	12.87 (9.16–18.08)	0.27 (0.15–0.50)
Wash hands after coming inside								
Always	24	0.14 (0.10–0.21)	4.80 (3.41–6.76)	0.63 (0.43–0.92)	0.06 (0.04–0.08)	1.84 (1.36–2.48)	9.73 (7.19–13.17)	0.14 (0.09–0.23)
Not always	26	0.12 (0.09–0.17)	5.16 (3.65–7.30)	0.49 (0.35–0.70)	0.05 (0.04–0.07)	1.27 (0.93–1.72)	10.63 (7.53–15.00)	0.15 (0.09–0.25)
Use of pesticides around the home								
No	30	0.13 (0.09–0.19)	4.73 (3.43–6.54)	0.51 (0.37–0.70)	0.06 (0.04–0.07)	1.44 (1.12–1.87)	9.87 (7.25–13.45)	0.13 (0.08–0.21)
Yes	20	0.13 (0.10–0.18)	5.39 (3.74–7.77)	0.62 (0.40–0.97)	0.06 (0.04–0.08)	1.62 (1.09–2.42)	10.68 (7.59–15.04)	0.17 (0.10–0.29)
Shoes worn inside home								
No	26	0.13 (0.09–0.19)	5.42 (3.93–7.48)	0.48 (0.33–0.68)	0.06 (0.04–0.07)	1.31 (0.95–1.81)	8.25 (5.93–11.46)	0.14 (0.09–0.22)
Yes	24	0.14 (0.10–0.19)	4.56 (3.16–6.57)	0.65 (0.45–0.94)	0.06 (0.04–0.08)	1.77 (1.33–2.36)	12.81 (9.56–17.17)	0.15 (0.09–0.25)
Dog/cat/pet in the house								
No	31	0.12 (0.09–0.16)	4.81 (3.46–6.68)	0.51 (0.36–0.71)	0.05 (0.04–0.07)	1.58 (1.20–2.07)	9.33 (6.82–12.78)	0.14 (0.09–0.21)
Yes	19	0.16 (0.10–0.25)	5.29 (3.74–7.50)	0.63 (0.42–0.95)	0.06 (0.05–0.08)	1.42 (0.97–2.07)	11.75 (8.62–16.02)	0.16 (0.10–0.28)
	n	*Manganese*	*Nickel*	*Selenium*	*Strontium*	*Thallium*	*Vanadium*	*Zinc*
Played with metal jewelry								
No	28	0.10 (0.07–0.13)	0.24 (0.16–0.38)	53.70 (42.82–67.35)	32.26 (22.92–45.41)	0.10 (0.07–0.13)	0.08 (0.06–0.11)	153.1 (118.6–197.5)
Yes	22	0.10 (0.07–0.13)	0.30 (0.18–0.48)	53.60 (40.99–70.09)	36.43 (23.89–55.56)	0.11 (0.08–0.15)	0.09 (0.07–0.12)	163.8 (113.5–236.3)
Played with painted toys								
No	31	0.10 (0.08–0.13)	0.26 (0.17–0.41)	53.58 (42.17–68.08)	34.06 (24.41–47.54)	0.10 (0.08–0.14)	0.09 (0.07–0.11)	147.5 (113.0–192.6)
Yes	19	0.09 (0.06–0.14)	0.27 (0.16–0.45)	53.77 (42.71–67.71)	33.99 (21.76–53.09)	0.10 (0.07–0.13)	0.08 (0.06–0.12)	175.8 (123.0–251.2)
Played with cosmetics								
No	29	0.09 (0.06–0.12)	0.25 (0.16–0.41)	49.37 (39.51–61.68)	29.17 (20.97–40.57)	0.10 (0.08–0.13)	0.08 (0.06–0.10)	140.5 (108.7–181.5)
Yes	21	0.12 (0.09–0.15)	0.28 (0.18–0.44)	60.19 (46.15–78.52)	42.12 (27.52–64.47)	0.10 (0.07–0.14)	0.10 (0.07–0.13)	184.9 (129.1–264.9)
Played with/wore sunscreen								
No	19	0.14 (0.11–0.20)	0.34 (0.18–0.64)	64.04 (46.40–88.40)	39.46 (26.22–59.38)	0.14 (0.10–0.18)	0.12 (0.08–0.16)	228.7 (159.4–328.2)
Yes	31	0.08 (0.06–0.10)	0.23 (0.16–0.33)	48.14 (39.88–58.10)	31.09 (22.01–43.91)	0.08 (0.06–0.11)	0.07 (0.06–0.09)	125.5 (99.8–157.9)
Have piercings								
No	28	0.09 (0.06–0.12)	0.30 (0.19–0.49)	52.88 (42.31–66.10)	28.94 (21.25–39.41)	0.10 (0.08–0.14)	0.08 (0.06–0.11)	145.3 (110.6–190.8)
Yes	22	0.11 (0.08–0.15)	0.23 (0.15–0.34)	54.65 (41.61–71.79)	41.84 (26.74–65.45)	0.10 (0.07–0.14)	0.10 (0.07–0.13)	175.0 (124.6–245.8)
Played with crayons								
No	7	0.09 (0.04–0.20)	0.21 (0.10–0.45)	46.39 (23.56–91.35)	42.97 (24.12–76.56)	0.08 (0.04–0.17)	0.09 (0.05–0.18)	147.7 (71.3–305.9)
Yes	43	0.10 (0.08–0.12)	0.28 (0.19–0.40)	54.94 (46.14–65.41)	32.77 (24.51–43.81)	0.11 (0.08–0.13)	0.09 (0.07–0.11)	159.4 (127.3–199.5)
Played with artist paint								
No	23	0.10 (0.07–0.14)	0.29 (0.17–0.48)	51.52 (39.43–67.31)	33.04 (22.01–49.60)	0.09 (0.07–0.12)	0.09 (0.07–0.12)	157.5 (114.0–217.5)
Yes	27	0.09 (0.07–0.13)	0.25 (0.16–0.39)	55.54 (44.35–69.56)	34.91 (24.52–49.70)	0.11 (0.08–0.15)	0.08 (0.06–0.11)	157.9 (118.3–210.6)
Played with ceramics								
No	45	0.10 (0.08–0.12)	0.26 (0.18–0.36)	54.45 (45.53–65.12)	32.34 (24.59–42.53)	0.11 (0.09–0.13)	0.09 (0.07–0.11)	159.7 (128.1–199.2)
Yes	5	0.09 (0.02–0.36)	0.35 (0.10–1.25)	47.01 (23.93–92.32)	53.91 (20.43–142.22)	0.06 (0.03–0.16)	0.09 (0.03–0.27)	140.1 (55.0–358.3)
Frequency playing outside (hrs/wk)								
Never to <2.5	15	0.10 (0.07–0.13)	0.16 (0.10–0.27)	52.42 (36.92–74.44)	32.15 (18.23–56.71)	0.09 (0.06–0.12)	0.08 (0.06–0.12)	173.1 (118.0–253.9)
2.5 to <5	16	0.07 (0.05–0.11)	0.22 (0.14–0.36)	48.13 (35.10–65.99)	23.95 (15.40–37.23)	0.09 (0.06–0.13)	0.07 (0.05–0.10)	153.2 (103.9–225.8)
≥5	19	0.13 (0.09–0.18)	0.45 (0.25–0.83)	59.89 (45.77–78.35)	47.86 (32.84–69.73)	0.13 (0.09–0.19)	0.11 (0.08–0.15)	150.1 (102.5–219.7)
Wash hands after coming inside								
Always	24	0.10 (0.07–0.14)	0.30 (0.17–0.52)	59.80 (47.90–74.67)	32.93 (23.20–46.73)	0.12 (0.09–0.15)	0.09 (0.07–0.12)	164.4 (123.6–218.6)
Not always	26	0.10 (0.07–0.13)	0.24 (0.16–0.35)	48.54 (37.60–62.67)	35.09 (23.55–52.27)	0.09 (0.06–0.13)	0.08 (0.06–0.13)	151.7 (110.4–208.6)
Use of pesticides around the home								
No	30	0.09 (0.07–0.12)	0.24 (0.16–0.37)	50.22 (39.71–63.51)	30.28 (20.83–44.02)	0.11 (0.08–0.14)	0.08 (0.06–0.10)	142.2 (105.4–191.9)
Yes	20	0.11 (0.08–0.14)	0.31 (0.18–0.52)	59.26 (46.50–75.51)	40.55 (28.85–57.00)	0.09 (0.07–0.13)	0.10 (0.07–0.13)	184.1 (139.7–242.7)
Shoes worn inside home								
No	26	0.08 (0.06–0.11)	0.29 (0.18–0.46)	48.40 (38.33–61.12)	28.27 (19.50–40.99)	0.09 (0.06–0.11)	0.08 (0.06–0.11)	147.5 (111.9–194.5)
Yes	24	0.12 (0.08–0.16)	0.24 (0.15–0.39)	59.99 (46.78–76.93)	41.62 (28.89–59.95)	0.12 (0.09–0.16)	0.09 (0.07–0.13)	169.5 (121.7–236.0)
Dog/cat/pet in the house								
No	31	0.09 (0.07–0.12)	0.27 (0.17–0.44)	53.86 (42.86–67.67)	28.42 (20.02–40.35)	0.09 (0.07–0.13)	0.08 (0.06–0.10)	169.7 (128.2–224.7)
Yes	19	0.11 (0.08–0.16)	0.25 (0.17–0.37)	53.33 (41.09–69.21)	45.66 (31.78–65.61)	0.11 (0.08–0.15)	0.10 (0.08–0.14)	139.9 (101.3–193.3)

a In the 3 months before study participation.

**Table 6.** Geometric Mean (95% Confidence Interval) of Urinary Metal Concentrations (μg/g Creatinine) by Average Frequency of Consumption of Selected Foods in the Last 3 Months^a^ Among 50 Children, Ages 5–12, Children’s Health and Research on Metals Study, Houston, TX, August–November 2019

	n	*Antimony*	*Arsenic*	*Barium*	*Cadmium*	*Copper*	*Iron*	*Lead*
Fruit juice								
Less than daily	31	0.15 (0.11–0.21)	4.48 (3.35–6.00)	0.61 (0.46–0.82)	0.06 (0.05–0.07)	1.59 (1.29–1.97)	10.82 (8.19–14.29)	0.15 (0.10–0.24)
At least daily	18	0.10 (0.07–0.15)	5.85 (3.75–9.12)	0.46 (0.27–0.77)	0.05 (0.04–0.07)	1.36 (0.82–2.25)	8.51 (5.79–12.51)	0.12 (0.08–0.17)
Milk								
Less than daily	25	0.17 (0.11–0.24)	6.31 (4.51–8.84)	0.65 (0.48–0.89)	0.07 (0.05–0.09)	1.56 (1.12–2.17)	12.10 (8.70–16.82)	0.25 (0.15–0.44)
At least daily	25	0.10 (0.07–0.14)	3.94 (2.85–5.45)	0.47 (0.31–0.70)	0.05 (0.04–0.06)	1.47 (1.09–1.98)	8.58 (6.31–11.67)	0.08 (0.06–0.11)
Grains								
Less than daily	29	0.15 (0.11–0.22)	4.94 (3.58–6.83)	0.72 (0.54–0.95)	0.06 (0.05–0.08)	1.66 (1.26–2.18)	11.29 (8.44–15.09)	0.16 (0.10–0.24)
At least daily	21	0.10 (0.07–0.15)	5.05 (3.48–7.32)	0.39 (0.25–0.60)	0.05 (0.03–0.06)	1.33 (0.92–1.92)	8.85 (6.13–12.77)	0.13 (0.08–0.23)
Fruits								
Less than daily	15	0.14 (0.09–0.21)	4.04 (2.56–6.36)	0.44 (0.31–0.63)	0.05 (0.04–0.07)	1.12 (0.83–1.53)	9.14 (6.41–13.02)	0.11 (0.06–0.21)
At least daily	35	0.13 (0.09–0.17)	5.46 (4.11–7.25)	0.61 (0.44–0.85)	0.06 (0.05–0.08)	1.72 (1.31–2.26)	10.68 (7.99–14.26)	0.16 (0.11–0.25)
Vegetables								
Less than daily	28	0.14 (0.10–0.19)	4.64 (3.36–6.41)	0.57 (0.41–0.79)	0.06 (0.04–0.07)	1.40 (1.04–1.90)	10.63 (7.98–14.17)	0.14 (0.09–0.22)
At least daily	22	0.12 (0.08–0.18)	5.46 (3.78–7.89)	0.53 (0.35–0.82)	0.06 (0.04–0.08)	1.67 (1.21–2.29)	9.65 (6.62–14.06)	0.15 (0.09–0.26)
Rice								
Less than daily	38	0.13 (0.10–0.17)	4.57 (3.47–6.01)	0.54 (0.41–0.72)	0.05 (0.04–0.07)	1.41 (1.11–1.79)	9.52 (7.38–12.26)	0.13 (0.09–0.19)
At least daily	12	0.13 (0.07–0.23)	6.59 (4.05–10.72)	0.59 (0.32–1.09)	0.06 (0.04–0.09)	1.89 (1.13–3.18)	12.65 (7.54–21.22)	0.21 (0.10–0.41)
Mexican candy								
Less than weekly	34	0.12 (0.09–0.16)	4.25 (3.19–5.66)	0.52 (0.38–0.72)	0.05 (0.04–0.06)	1.31 (1.01–1.71)	8.80 (6.75–11.47)	0.12 (0.08–0.19)
At least weekly	16	0.16 (0.10–0.26)	7.01 (4.69–10.48)	0.62 (0.39–0.98)	0.08 (0.06–0.11)	2.04 (1.41–2.94)	13.92 (9.28–20.88)	0.20 (0.11–0.38)
Red meat								
Once per week or less	24	0.11 (0.07–0.16)	4.25 (2.97–6.08)	0.58 (0.39–0.87)	0.05 (0.04–0.07)	1.54 (1.08–2.20)	9.39 (6.63–13.28)	0.12 (0.07–0.20)
>Once per week	26	0.16 (0.11–0.22)	5.78 (4.20–7.97)	0.53 (0.38–0.74)	0.06 (0.05–0.08)	1.49 (1.13–1.96)	10.99 (8.10–14.90)	0.17 (0.11–0.27)
Chicken or pork								
Once per week or less	17	0.16 (0.09–0.27)	4.72 (2.98–7.47)	0.47 (0.30–0.75)	0.06 (0.04–0.09)	1.51 (1.01–2.24)	9.31 (6.16–14.07)	0.15 (0.07–0.30)
>Once per week	33	0.12 (0.09–0.16)	5.13 (3.86–6.83)	0.60 (0.44–0.82)	0.06 (0.05–0.07)	1.52 (1.16–1.98)	10.67 (8.09–14.08)	0.15 (0.10–0.21)
Fish/shellfish								
Less than weekly	30	0.15 (0.10–0.21)	5.57 (4.09–7.59)	0.71 (0.52–0.98)	0.06 (0.05–0.08)	1.69 (1.30–2.21)	11.79 (8.79–15.82)	0.19 (0.11–0.31)
At least weekly	20	0.11 (0.08–0.15)	4.22 (2.87–6.20)	0.38 (0.26–0.56)	0.04 (0.03–0.06)	1.28 (0.88–1.85)	8.18 (5.78–11.59)	0.10 (0.07–0.15)
Multivitamin use								
No	40	0.12 (0.10–0.16)	5.10 (3.88–6.69)	0.54 (0.40–0.72)	0.06 (0.04–0.07)	1.51 (1.19–1.92)	10.42 (8.15–13.32)	0.14 (0.10–0.21)
Yes	10	0.16 (0.06–0.39)	4.57 (2.63–7.92)	0.62 (0.33–1.16)	0.06 (0.04–0.10)	1.51 (0.83–2.76)	9.33 (4.97–17.53)	0.17 (0.08–0.37)
	n	Manganese	Nickel	Selenium	Strontium	Thallium	Vanadium	Zinc
Fruit juice								
Less than daily	31	0.10 (0.07–0.13)	0.27 (0.18–0.41)	52.70 (42.52–65.33)	37.02 (27.32–50.17)	0.11 (0.08–0.15)	0.09 (0.07–0.12)	167.5 (130.4–215.2)
At least daily	18	0.09 (0.06–0.13)	0.23 (0.14–0.39)	54.04 (39.80–73.36)	28.16 (16.86–47.03)	0.08 (0.06–0.11)	0.08 (0.05–0.11)	135.1 (91.1–200.5)
Milk								
Less than daily	25	0.12 (0.09–0.16)	0.37 (0.22–0.63)	57.78 (44.42–75.17)	42.08 (29.84–59.32)	0.11 (0.08–0.16)	0.10 (0.07–0.13)	174.5 (125.5–242.7)
At least daily	25	0.08 (0.06–0.11)	0.19 (0.14–0.27)	49.82 (39.94–62.14)	27.53 (18.65–40.63)	0.09 (0.08–0.11)	0.08 (0.06–0.10)	142.5 (108.8–186.6)
Grains								
Less than daily	29	0.10 (0.08–0.14)	0.34 (0.22–0.53)	54.66 (43.26–69.05)	40.53 (29.63–55.45)	0.11 (0.08–0.15)	0.10 (0.08–0.13)	173.7 (131.7–229.8)
At least daily	21	0.09 (0.05–0.12)	0.19 (0.12–0.30)	52.30 (40.51–67.52)	26.74 (17.14–41.72)	0.09 (0.07–0.12)	0.07 (0.05–0.10)	138.0 (99.6–191.2)
Fruits								
Less than daily	15	0.08 (0.06–0.11)	0.18 (0.10–0.32)	47.38 (33.91–66.21)	23.90 (14.79–38.64)	0.09 (0.06–0.14)	0.07 (0.05–0.10)	153.7 (103.3–228.5)
At least daily	35	0.10 (0.08–0.14)	0.29 (0.20–0.44)	56.59 (46.36–69.08)	39.60 (29.16–53.78)	0.11 (0.08–0.13)	0.09 (0.07–0.12)	159.4 (123.4–206.0)
Vegetables								
Less than daily	28	0.10 (0.07–0.12)	0.27 (0.16–0.43)	54.87 (43.33–69.49)	32.77 (23.08–46.52)	0.10 (0.07–0.13)	0.08 (0.06–0.11)	169.8 (129.1–223.5)
At least daily	22	0.10 (0.07–0.15)	0.27 (0.17–0.41)	52.14 (40.48–67.17)	35.72 (23.68–53.88)	0.11 (0.08–0.15)	0.09 (0.07–0.13)	143.5 (102.3–201.1)
Rice								
Less than daily	38	0.09 (0.07–0.11)	0.22 (0.16–0.32)	52.03 (43.08–62.84)	31.67 (23.24–43.16)	0.10 (0.08–0.13)	0.08 (0.06–0.10)	150.1 (117.4–192.0)
At least daily	12	0.14 (0.08–0.23)	0.46 (0.23–0.92)	59.14 (39.30–88.98)	42.75 (26.17–69.83)	0.11 (0.08–0.16)	0.12 (0.07–0.19)	184.2 (119.8–283.1)
Mexican candy								
Less than weekly	34	0.09 (0.07–0.11)	0.22 (0.16–0.32)	49.21 (40.27–60.13)	29.30 (21.25–40.40)	0.08 (0.07–0.11)	0.07 (0.06–0.09)	139.3 (110.7–175.4)
At least weekly	16	0.13 (0.09–0.19)	0.38 (0.20–0.72)	64.48 (47.09–88.29)	46.79 (30.38–72.06)	0.15 (0.10–0.21)	0.13 (0.09–0.18)	205.1 (132.5–317.6)
Red meat								
Once per week or less	24	0.10 (0.07–0.14)	0.23 (0.13–0.38)	48.60 (37.41–63.12)	32.92 (22.38–48.42)	0.09 (0.07–0.12)	0.08 (0.06–0.11)	145.1 (105.1–200.4)
>Once per week	26	0.10 (0.07–0.13)	0.31 (0.17–0.43)	58.79 (47.04–73.48)	35.10 (24.25–50.80)	0.11 (0.08–0.15)	0.09 (0.07–0.12)	170.2 (128.2–226.1)
Chicken or pork								
Once per week or less	17	0.09 (0.06–0.13)	0.23 (0.11–0.48)	52.14 (37.01–73.44)	29.81 (18.11–49.07)	0.11 (0.07–0.17)	0.08 (0.05–0.11)	151.5 (101.1–226.9)
> Once per week	33	0.10 (0.08–0.13)	0.29 (0.21–0.40)	54.45 (44.76–66.24)	36.44 (26.68–49.76)	0.10 (0.08–0.12)	0.09 (0.07–0.12)	161.0 (125.1–207.1)
Fish/shellfish								
Less than weekly	30	0.11 (0.08–0.14)	0.35 (0.22–0.54)	61.22 (49.61–75.57)	42.20 (31.44–56.65)	0.12 (0.09–0.16)	0.10 (0.08–0.13)	164.2 (123.4–218.3)
At least weekly	20	0.09 (0.06–0.12)	0.18 (0.12–0.27)	44.02 (33.56–57.73)	24.65 (15.49–39.22)	0.08 (0.06–0.10)	0.07 (0.05–0.10)	148.4 (107.4–205.1)
Multivitamin use								
No	40	0.10 (0.08–0.12)	0.27 (0.19–0.39)	54.59 (45.41–65.63)	33.59 (24.96–45.21)	0.10 (0.08–0.13)	0.09 (0.07–0.11)	175.6 (138.8–222.3)
Yes	10	0.10 (0.05–0.21)	0.24 (0.11–0.52)	50.05 (31.23–80.22)	35.86 (19.47–66.03)	0.11 (0.07–0.18)	0.09 (0.05–0.15)	102.4 (69.7–150.6)

a Prior to the interview.
