# Supplementary material for: Assessing Metal Exposures Among Children Living in Environmental Justice Communities Near Metal Recycling Facilities in Houston, Texas
Source: Environ Justice. Author manuscript; Available in PMC 2025 Jul 28. (PMC12302942; doi:10.1089/env.2022.0023)
Supplement: Env Justice 2014 17:2, 128-142 (supplementary material) [file NIHMS2027570-supplement-Env_Justice_2014_17_2__128-142__supplementary_material_.docx]

**Supplemental Table 1**. Spearman correlation of urinary metal concentrations (µg/g creatinine) among 50 children ages 5-12, Children’s Health and Research on Metals (CHaRM) Study, Houston, Texas, August–November 2019. ^*^

|  | Antimony | Arsenic | Barium | Cadmium | Copper | Iron | Lead | Manganese | Nickel | Selenium | Strontium | Thallium | Vanadium | Zinc |
| --- | --- | --- | --- | --- | --- | --- | --- | --- | --- | --- | --- | --- | --- | --- |
| Antimony | 1.000 |  |  |  |  |  |  |  |  |  |  |  |  |  |
| Arsenic | 0.442 | 1.000 |  |  |  |  |  |  |  |  |  |  |  |  |
| Barium | 0.354 | 0.173 | 1.000 |  |  |  |  |  |  |  |  |  |  |  |
| Cadmium | 0.658 | 0.637 | 0.476 | 1.000 |  |  |  |  |  |  |  |  |  |  |
| Copper | 0.454 | 0.327 | 0.626 | 0.539 | 1.000 |  |  |  |  |  |  |  |  |  |
| Iron | 0.577 | 0.426 | 0.674 | 0.696 | 0.704 | 1.000 |  |  |  |  |  |  |  |  |
| Lead | 0.738 | 0.596 | 0.359 | 0.571 | 0.391 | 0.563 | 1.000 |  |  |  |  |  |  |  |
| Manganese | 0.519 | 0.367 | 0.724 | 0.610 | 0.682 | 0.836 | 0.568 | 1.000 |  |  |  |  |  |  |
| Nickel | 0.355 | 0.555 | 0.405 | 0.574 | 0.497 | 0.490 | 0.571 | 0.452 | 1.000 |  |  |  |  |  |
| Selenium | 0.679 | 0.612 | 0.491 | 0.670 | 0.711 | 0.690 | 0.621 | 0.525 | 0.556 | 1.000 |  |  |  |  |
| Strontium | 0.417 | 0.294 | 0.831 | 0.567 | 0.537 | 0.826 | 0.424 | 0.740 | 0.356 | 0.516 | 1.000 |  |  |  |
| Thallium | 0.634 | 0.579 | 0.446 | 0.645 | 0.720 | 0.696 | 0.568 | 0.608 | 0.417 | 0.741 | 0.447 | 1.000 |  |  |
| Vanadium | 0.527 | 0.371 | 0.740 | 0.645 | 0.725 | 0.805 | 0.524 | 0.890 | 0.482 | 0.535 | 0.737 | 0.672 | 1.000 |  |
| Zinc | 0.517 | 0.585 | 0.342 | 0.701 | 0.516 | 0.596 | 0.492 | 0.502 | 0.554 | 0.678 | 0.404 | 0.541 | 0.543 | 1.000 |

^*^P<0.05 for spearman correlations between all urinary metals except between Arsenic and Barium.

Chromium (Cr) and cobalt (Co) were excluded for the correlation analyses due to 74 and 62 percent of samples not detected, respectively.
